# Thyroid Cell Differentiation from Murine Induced Pluripotent Stem Cells

**DOI:** 10.3389/fendo.2015.00056

**Published:** 2015-04-22

**Authors:** Risheng Ma, Syed A. Morshed, Rauf Latif, Terry F. Davies

**Affiliations:** ^1^Thyroid Research Unit, Department of Medicine, James J. Peters Veterans Affairs Medical Center, Icahn School of Medicine at Mount Sinai, New York, NY, USA

**Keywords:** induced pluripotent stem cells, thyroid differentiation, PAX8, NKX2-1, TSH receptor

## Abstract

**Background:**

Here, we demonstrate the successful differentiation of induced pluripotent stem (iPS) cells into functional thyroid cells indicating the therapeutic potential of this approach when applied to individuals with thyroid deficiency.

**Research design and methods:**

Using embryonic murine fibroblasts, we generated iPS cells with a single lentiviral “stem cell cassette” vector and then differentiated these iPS cells into thyroid cells after transfection with PAX8 and NKX2-1 by Activin A and TSH stimulation.

**Results:**

The generated iPS cells expressed pluripotent stem cell markers as assessed using both reverse transcription quantitative PCRs and immunofluorescence staining with ~0.5% reprograming efficiency. Compared to control cells, the expression of thyroid-specific genes NIS, TSHR, Tg, and TPO were greatly enhanced in PAX8^+^NKX2-1^+^ iPS cells after differentiation. On stimulation with TSH, these differentiated iPS cells were also capable of dose-dependent cAMP generation and radioiodine uptake indicative of functional thyroid epithelial cells. Furthermore, the cells formed three-dimensional follicles in culture, and “thyroid organoids” formed after PAX8^+^NKX2-1^+^ iPS cells transplanted into nude mice, and all expressed Tg protein as judged immunohistochemically. Taken together, thyroid epithelial cells differentiated from iPS cells, which were themselves derived from murine fibroblasts, exhibited very similar properties to thyroid cells previously developed from traditional murine embryonic stem cells.

**Conclusion:**

Thyroid cells differentiated from iPS cells offer the opportunity to examine the detailed transcriptional regulation of thyroid cell differentiation and may provide a useful future source for individualized regenerative cell therapy.

## Introduction

Thyroid deficiency, as an irreversible change, occurs due to autoimmune disease (Hashimoto’s thyroiditis), radioiodine therapy, and thyroid surgery. Although the majority of hypothyroid patients feel well on thyroid hormone replacement therapy, there remains a small proportion claiming not to feel normal. An improved treatment strategy for these patients is still needed. Although differentiated human pluripotent embryonic stem (ES) cells are the ideal seed cells for transplantation, ethical issues regarding their source and potential immunological barriers have restricted their application in cell therapy. With the advances in stem cell biology and the recent discovery that somatic mammalian cells can be epigenetically reprogramed to a pluripotent state through the exogenous expression of the four transcription factors – “yamanaka factors” – has opened up the field of personalized regenerative medicine (1, 2). The expression of these four defined factors – OCT3/4, SOX2, KLF4, cMYC – into an adult somatic cell has the ability to dedifferentiate it into an embryonic cell with potential of developing into any cell lineages under appropriate environmental cues. Thus, IPSC holds great promise as an unlimited source for individualized cell replacement therapy (1) without the ethical or practical concerns associated with ES cells (3). Nevertheless, there remain technical issues with this technology due to lack of full understanding of the epigenetic changes during the reprograming process and the challenge of optimizing the reprograming efficiency within each tissue type (4).

We, and others, have recently described the induction of thyroid follicular cell differentiation from human and mouse ES cells by over-expression of the thyroid transcription factors PAX8 and NKX2-1 (5–7). In the present study, we generated murine induced pluripotent stem (iPS) cells using a single lentiviral “stem cell cassette” (STEMCCA) vector and fully characterized them prior to over-expression of the two critical transcription factors – PAX8 and NKX2-1. Further, these double transfected cells, as described previously (5), were induced to form endoderm-derived embryoid bodies (EBs) and finally differentiated into thyroid follicles. Our results indicate that the thyroid cells generated from iPS cells exhibited identical properties to those cells derived from ES cells confirming that iPS cells may be useful source for cell therapy and also as a tool for deriving patient-specific human cell lines for studying thyroid cell biology.

## Materials and Methods

### Generation and maintenance of mouse induced pluripotent stem cell

Mouse iPS cells were generated by reprograming mouse embryonic fibroblasts (MEFs) (originally derived from the Swiss Webster mouse and supplied by the Pluripotent Stem Cell Shared Resource Facility at Mount Sinai) using a single lentiviral vector, expressing a “stem cell cassette” composed of the four transcription factors, Oct4, Klf4, Sox2, and cMyc from a single multicistronic transcript (8). About 100,000 fibroblasts/well were plated into 0.1% gelatin-coated 6-well plates and infected with 15 μl of concentrated virus in the presence of polybrene (5 μg/ml) after the cells had attached. After 16 h, the medium was replaced with mouse ES cell culture medium [DMEM supplemented with 15% FBS, l-glutamine, penicillin/streptomycin, non-essential amino acids, β-mercaptoethanol, and 1,000 U/ml leukemia inhibitory factor (LIF)] and changed every other day. iPS colonies were picked 15–20 days post-infection on the basis of morphology and expanded by plating on irradiation-treated MEFs in ES cell culture medium. After one to two passages, iPS cells were directly adapted to feeder-free culture conditions.

### Generation of PAX8, or NKX2-1, or PAX8 + NKX2-1 expressing iPS cell lines

Two vectors, M48EF1αPAX8IVREShyg and M48EF1αNKX2-1IVRESneo, were kindly provided by Dr. Uwe Haberkorn of the German Cancer Research Center, Heidelberg, Germany (8). These vectors, alone or together, were electroporated into iPS cells (Neon transfection system, Life technologies) and after 2 days, the cells were cultured in either hygromycin (0.5 mg/ml) or G418 (0.8 mg/ml), or both for at least 4 weeks until resistant clones were established. Stable clones were then selected and characterized for their gene expression and for further differentiation.

### Differentiation of iPS cells

Embryoid bodies were differentiated as described previously (9). In brief, single ES cell suspensions were plated in ultralow attachment dishes (Costar 3471, Corning Inc., NY, USA) to induce EB formation in the absence of LIF. EBs were cultured for 1 day in the same culture medium as ES cells except for the lack of LIF. The following day, EBs were harvested and allowed to settle by gravity in a 50-ml tube and transferred to new dishes and cultured in fresh medium supplemented with or without 50 ng/ml human Activin A (R&D Systems, Inc.) for 5 days. To induce the differentiation of iPS cells into thyroid cells, EBs were collected and embedded in growth factor-restricted Matrigel (BD Biosciences) and placed into six-well plates in differentiation medium that contained DMEM supplemented with penicillin/streptomycin, 15% KnockOut serum replacement medium (Invitrogen Life Technologies), 5% protein-free hybridoma medium (PFHM-II; Invitrogen Life Technologies), 1.5 × 10^−4^M monothioglycerol, and 1,000 μU/ml human recombinant TSH (Fitzgerald Industries, Concord, MA, USA) for another 16 days. Cells were harvested for analysis at days 5 and 21 (total time) of culture.

### Gene expression analysis

For gene expression analysis, total RNA was extracted using RNeasy (Qiagen Ltd.) and treated with ribonuclease-free deoxyribonuclease (Qiagen). Five micrograms of total RNA were reverse transcribed into cDNA using the SuperScript III system (Invitrogen). Real-time quantitative RT-PCR (qRT-PCR) was carried out using SYBR green qPCR master mix (Applied Biosystems) employing the StepOnePlus system (Applied Biosystems). Relative expression levels of each gene in real-time were analyzed using the 2^−ΔΔCT^ method and normalized to the expression of the housekeeping gene GAPDH. Data presented (mean) are from three independent experiments in which all sample sets were analyzed in triplicate.

### Immunofluorescence staining

Cells were grown in Delta T culture dishes and fixed with 4% paraformaldehyde in phosphate-buffered saline (PBS) and rinsed three times with PBS. After being permeabilized in methanol at −20°C for 10 min, the cells were blocked for 60 min with 5% normal goat serum in PBS. Cells were then incubated with appropriate primary antibodies in 0.1% Tween 20 and 1% BSA in PBS overnight at 4°C. Cells were rinsed three times with PBS and incubated with the appropriate secondary antibody. Then cells were rinsed three times with PBS, and mounted using hard set mounting media containing DAPI (Vector Laboratories). Fluorescence micrographs were acquired using a LSM 700 confocal microscope with a 63× objective, and images were processed and assembled in Photoshop CS (Adobe, San Jose, CA, USA).

### TSHR functional assessment

Intracellular cAMP generation was measured with a cAMP enzyme immunoassay kit (GE Healthcare Bio-Sciences). Briefly, the differentiated iPS cells were seeded in a 24-well tissue culture plate at a density of ~1.0 × 10^5^ cells/dish in 1 ml medium 24 h prior to the experiment. The cells were then stimulated with increasing concentrations of TSH for 1 h. The medium was aspirated, the cells washed with fresh PBS and lysed for 10 min. A total of 100 μl of each sample was transferred into the appropriate well of the immunoassay microtiter plate and intracellular cAMP was measured as described by the manufacturer.

### Radioactive iodine uptake

The differentiated iPS cells were seeded in a 24-well plate at a density of ~1.0 × 10^5^ cells/well for 24–48 h. Cells were then washed with modified Hanks’ balanced salt solution, then incubated in the same buffer containing 20 μM sodium iodide supplemented with 10 μCi carrier-free Na^125^I to give a specific activity of 10 μCi/mmol for 45 min at 37°C in a humidified atmosphere. Parallel wells of cells were incubated in the absence or presence of TSH and/or perchlorate. Aspirating the radioactive solution and washing twice with ice-cold Hanks’ balanced salt solution terminated the uptake reactions. To determine the amount of ^125^I-accumulated in the cells, 95% ethanol was added to each well for 20 min at 4°C, then quantitated in a γ-counter, as previously described (10). ^125^I-uptake was expressed as cpm per well per 45 min. The values represent the mean ± SEM of two independent experiments performed in triplicate.

### Thyroid follicle formation *in vivo*

Five-week-old nude male mice (The Jackson Laboratory) were housed in pathogen-free conditions with the approval of the Institutional Animal Care and Use Committee of Mount Sinai School of Medicine. The EB cells derived from transfected iPS cells were re-suspended in Matrigel-PBS and injected subcutaneously. The injected Matrigel-cells rapidly formed a solid structure. After 3–4 weeks, the formed structure were surgically excised from the mice without connective tissues and embedded into paraffin and sectioned for immunohistochemistry.

## Results

### Generation and characterization of induced pluripotent stem cells

**Figure 1:**
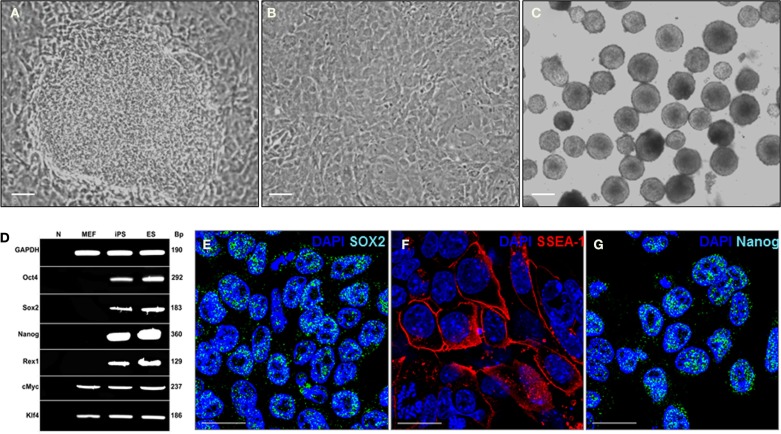
**Mouse iPS cells have cell morphology and staining characteristics of mouse ES cells**. **(A)** MEFs formed multilayered, tightly packed cells with well-defined borders after infection with the STEMCCA Lentivirus. This figure shows a representative phase contrast image of a single mouse iPS cell colony taken at 12 days post-infection. Scale = 200 μm. **(B)** Control well of MEFs not infected with the STEMCCA Lentivirus. Cells remain in a monolayer culture with no ES cell-like colonies observed. This is a representative phase contrast image taken at the same time point as that in **(A)**. Scale = 200 μm. **(C)** Embryoid bodies formed from iPS cells derived from MEFs. Scale = 200 μm. **(D)** Expression of ES cell marker genes detected by RT-PCR in iPS cells generated using the STEMCCA vector. **(E–G)** Representative immunostaining Sox2 **(E)**, SSEA-1 **(F)**, and Nanog **(G)** in the derived iPS cells. Scale = 100 μm. Abbreviations: N, negative control; MEF, mouse embryonic fibroblast; ES, mouse embryonic stem; iPS, induced pluripotent stem; SSEA-1, stage-specific embryonic antigen 1.

Induced pluripotent stem cells were derived from MEFs infected with the EF1a-STEMCCA Polycistronic (OKSM) Lentivirus (8). MEFs, reprogramed with the constitutive EF1a STEMCCA construct, showed a dramatic change in morphology already evident 6 days post-infection and formed colonies that displayed the typical morphology of ES cell colonies (Figure 1A). Control MEFs that had not been infected with the STEMCCA Lentivirus remained in a monolayer culture with no ES cell-like colonies observed (Figure 1B). The effective reprograming efficiency was ~0.5%, about 10-fold higher than that observed in prior reports (0.01–0.05%) (11, 12). The generated iPS cells were then able to spontaneously form EBs (Figure 1C). The molecular signature of the iPS cells was validated by RT-PCR and as expected showed expression of a variety of classical ES cell markers such as Oct4, Sox2, Nanog, and REX1, whereas, these genes were not expressed in the fibroblasts prior to reprograming (Figure 1D). Expression of ES cell markers including Sox2, SSAE-1, and Nanog are also illustrated using immunostaining (see Figures 1E–G). These data confirmed successful iPS reprograming from the MEF cells.

### Establishment of PAX8, NKX2-1, PAX8^+^NKX2-1^+^ iPS cell lines

**Figure 2:**
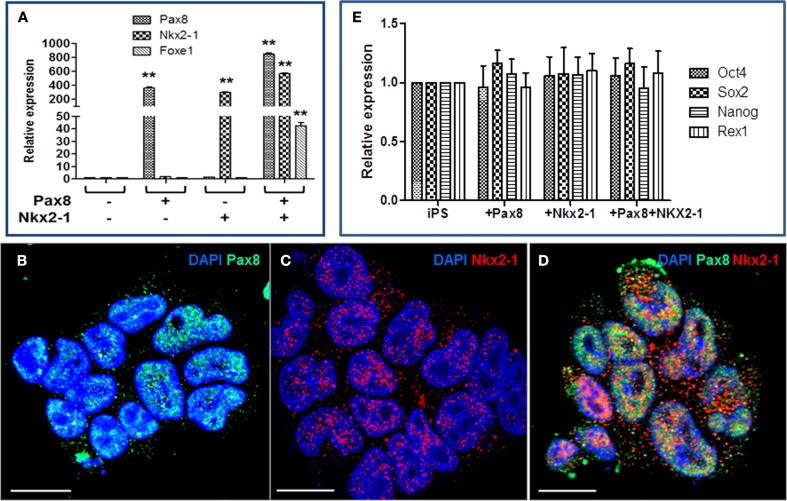
**Characterization of PAX8, NKX2-1, and Pax8^+^NKx2-1^+^ mouse iPS cell lines**. **(A)** RT-PCR analysis of selected iPS cell lines for thyroid transcription factors. The figure shows PAX8, NKX2-1, and FOXE1 transcription after culture in selection medium for up to 4 weeks. Data presented (mean) are from three independent experiments in which all sample sets were analyzed in triplicate. **p < 0.01 compared to untransfected iPS cells. **(B–D)** Immunofluorescence analysis of PAX8 and NKX2-1 in the transfected cell lines. Scale = 100 μm. **(E)** Retention of stemness in transfected cell lines is shown by the continued expression of Oct4, Sox2, Nanog, and Rex1 gene expression.

We have shown in previous studies that thyroid follicular cells can be generated from mouse ES cells by co-expressing thyroid transcription factors PAX8 and NKX2-1 and culturing the cells in the presence of activin A and TSH (5, 6). The generated iPS cells were, therefore, electroporated with the PAX8 and/or NKX2-1 vectors and selected with specific antibiotic to establish stable iPS cell lines (5). As evidenced by RT-PCR analysis, PAX8^+^ cells expressed high levels of PAX8, NKX2-1^+^ cells expressed high levels of NKX2-1, while, PAX8^+^NKX2-1^+^ cells expressed high levels of both PAX8 and NKX2-1 and FOXE1 expression was induced (Figure 2A). This was further confirmed by immunodetection of Pax8 and NKx2-1 nuclear expression in individual cell lines (Figures 2B–D). However, over-expression of these transcription factors at this stage did not change the pluripotent state of the iPS cells since they continued to express a variety of stem cell genes (Figure 2E). Although these iPS cell lines expressed a limited degree of thyroid-specific genes (TSHR, NIS, and Tg), Tg protein was not detected indicating that while co-expression of PAX8 and NKX2-1 in untreated iPS stable lines initiated a change in the cell’s fate, driving their differentiation toward a thyroid follicular cell lineage, the degree of differentiation was insufficient for thyrocyte fate determination.

### Differentiation of PAX8^+^NKX2-1^+^ iPS cells into thyroid cells

**Figure 3:**
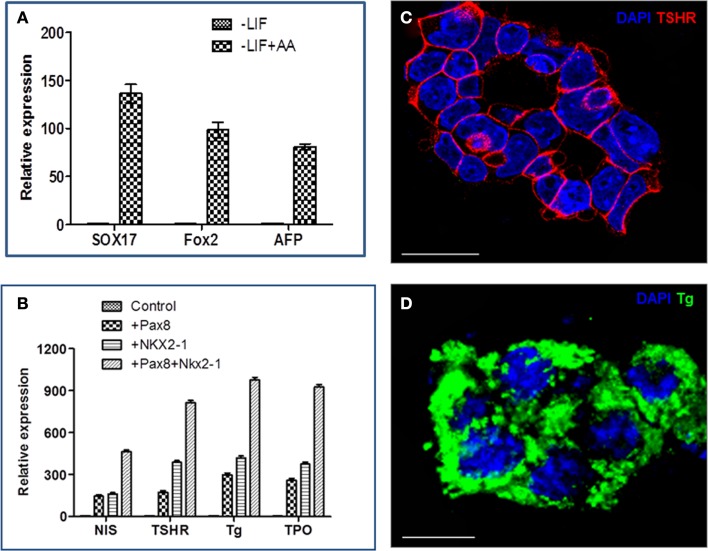
**The differentiation of iPS cells into thyroid cells**. **(A)** Representative RT-PCR analysis for the expression of endoderm markers, Sox17, Fox2, and AFP, in Activin A (AA) treated and untreated cells. Data were expressed as mean ± SEM and represent one of three separate experiments. **(B)** Thyroid-specific genes (Tg, TPO, TSHR, and NIS) in individual cell lines after differentiation with Activin A and TSH. Data were expressed as mean ± SEM and represent one of three separate experiments. The difference between control ES cells, Pax8^+^, Nkx2-1^+^ and double positive cells was highly significant (*p* < 0.01) for each of the mRNA examined. **(C,D)** Immunostaining of thyroid neofillicles derived from double transfected PAX8^+^NKX2-1^+^ iPS cells after differentiation with Activin A and TSH and showing TSHR expression (red) on the cell surface **(C)** and Tg expression (green) in the follicle lumen and cytoplasm **(D)**. Scale bar = 100 μm.

Individual iPS cells expressing PAX8, NKX2-1, or both factors were successfully differentiated into thyroid cells by Activin A and TSH as previously found for similar ES-derived cells (5). Since Activin A can induce endoderm differentiation (9), we cultured the double transfected cells in ultralow attachment dishes to encourage embryoid body formations (EBs) and treated these cells with Activin A for 5 days, resulting in the induction of endoderm markers Sox17, Fox2, and AFP (Figure 3A). These Activin A treated cells demonstrated upregulation of TSHR gene expression but no further induction of Tg transcription (5, 9). However, after further differentiation with TSH, thyroid-specific genes (NIS, TSHR, Tg, and TPO) were all better expressed in Pax8^+^NKX2-1^+^ iPS cells in comparison with single transfected iPS cell lines, as evidenced by qPCR analysis (Figure 3B). These differentiated double transfected cells formed three-dimensional thyroid follicle with widespread TSHR expression (Figure 3C) and Tg expression (Figure 3D), evidencing these cells as committed to their thyrocyte fate. We estimate that ~50% of cells formed thyroid follicles in these experiments.

### Functionality of differentiated thyroid cells

**Figure 4:**
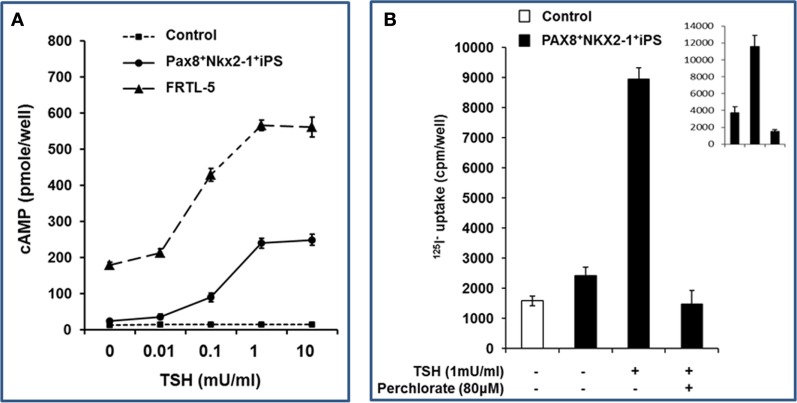
**Functionality of differentiated thyroid iPS cells**. **(A)** Differentiated Pax8^+^NKX2-1^+^ iPS cells demonstrated cAMP generation in response to increasing concentrations of TSH. The sensitivity of rat thyroid cells (FRTL-5) is illustrated for comparison within the same experiment. **(B)** Differentiated Pax8^+^NKX2-1^+^ iPS cells were exposed to ^125^I (as described in Section “Materials and Methods”) and the radioactive iodine uptake into the differentiated thyroid cells was measured with and without TSH. Specificity was shown by the use of sodium perchlorate to inhibit the action of NIS and prevent radioactive iodine uptake. The insert is the radioactive iodine uptake in rat thyroid cells, FRTL-5 cell.

The double transfected and differentiated iPS cells were further analyzed by assessment of TSH stimulated cAMP generation (Figure 4A). A dose-dependent cAMP response to TSH was observed with a sensitivity of 10 μU/ml; similar to the rat thyroid cell control. No response was seen in the control ES cells. These differentiated thyroid cells, maintained under steady-state conditions without TSH (as described in Section “Materials and Methods”), also demonstrated significantly increased ^125^I uptake in response to TSH which was inhibited by sodium perchlorate acting as a NIS inhibitor (Figure 4B).

### Differentiation of PAX8/NKX2-1 iPS cells into thyroid cells *in vivo*

**Figure 5:**
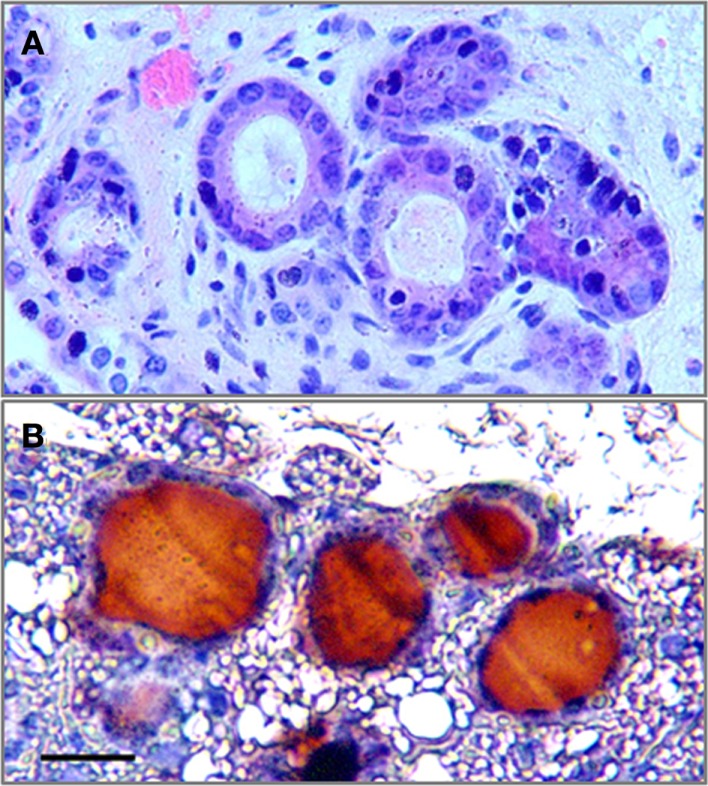
**The formation of thyroid follicles *in vivo***. **(A)** The histological analysis of Pax8^+^NKX2-1^+^ iPS cells grafted subcutaneously into nude mice after 4 weeks (H&E) showing follicle formation. **(B)** Immunostaining for Tg in the grafted tissue showed Tg deposition in the luminal compartment. Scale bar = 20 μm.

To assess the potential thyroid differentiation of iPS cells *in vivo*, the PAX8^+^NKX2-1^+^ iPS EB cells, following Activin A exposure for 5 days, were injected subcutaneously into 5-week-old nude mice. Histological analysis 4 weeks after transplantation demonstrated the formation of thyroid tissue in the host mice (Figure 5A) with Tg deposition in the follicular lumens (Figure 5B).

## Discussion

The exact mechanism of successful thyroid cell differentiation from stem cells is far from clear. It has previously been reported that murine and human ES cells can be induced to differentiate into functional thyroid follicles by the combined expression of Pax8 and Nkx2-1 using transfection or direct induction (5, 6). Here, we aimed to determine whether murine iPS cells would behave in a similar manner. We found that after differentiation with Activin A and TSH, Pax8^+^Nkx2-1^+^ expressing iPS cells were indeed induced to thyroid follicular cells in a similar manner to ES cells as evidenced by the expression of thyroid-specific genes and the formation of thyroid follicular-like structures *in vitro* and *in vivo*. iPS cells may, therefore, be an ideal source for cell replacement therapy when derived from hypothyroid patients.

We generated iPS cells from embryonic fibroblasts using a single lentiviral “stem cell cassette” (STEMCCA) vector, expressing the four transcription factors, Oct4, Klf4, Sox2, and cMyc, from a single multicistronic transcript which was highly efficient (8). These iPS cells were clearly pluripotent as shown by a number of accepted criteria such as their morphology and stem cell marker expression. iPS cells derived from mouse (11, 12) or human (1, 13) fibroblasts have been well demonstrated to offer the potential to replace many organs using readily accessible postnatal somatic cells and the use of a single lentiviral STEMCCA vector for the induction of iPS cells enabled high efficiency of reprograming and limited numbers of viral integrations, which is in marked contrast to previous reports using multiple vectors requiring >15 viral integrations (1, 12). Since the original discovery of iPS cells, there has been great progress in iPS cell research in improving both the efficiency and safety of the reprograming steps (14) and also in the differentiation of iPS cells for the treatment of several conditions (12, 15). However, the generation of patient-specific iPS cells is still a technical and time demanding procedure and long-term problems, such as cancer formation, are unlikely to be circumvented by this transfection approach. Direct chemical reprograming, therefore, is in need of further exploration (16). Epigenetic changes during the reprograming process have shown considerable differences between iPS and ES cells and this must also be addressed before iPS cell technology can be utilized therapeutically.

Tissue-specific transcription factors play a vital role in establishing cell identity during development. Tissue-specific gene expression reflects the coordinated activities of transcription factors that are restricted to one or a few cell types. Several thyroid-specific transcription factors have been identified and characterized, including Pax8, NKX2-1, Foxe1, and Hex (17, 18). Each of these factors controls the maintenance of the expression of others. For example, the simultaneous presence of Pax8, Nkx2-1, and Hex are required for the expression of Foxe1 (19, 20) and in this study, the expression of Foxe1 was also significantly induced in Pax8^+^Nkx2-1^+^ double transfected iPS cells in comparison to the control and single transfected iPS cells. These transcription factors have a central function in other embryonic tissues, but it is only in the endoderm cells committed to a thyroid cell fate that the combination of all four can be found. While HEX and FOXE1 are expressed throughout the endoderm, NKX2-1 and PAX8 expression is restricted to the thyroid placode indicating their crucial role in thyroid cell speciation. Here, we demonstrated that fibroblast derived iPS cells could also be induced to differentiate into thyroid follicular cells by over expressing Pax8 and Nkx2-1 in a similar manner to mouse ES cells with an efficiency approaching 50% of cells.

In summary, we showed the differentiation of mouse iPS cells into thyroid follicular cells via over-expression of PAX8 and NKX2-1 and induction with Activin A and TSH. These differentiated PAX8^+^NKX2-1^+^ expressing iPS cells expressed thyroid-specific genes and proteins, formed three-dimensional follicles when cultured with an extracellular matrix and formed Tg expressing thyroid follicles after transplantation into nude mice. This study demonstrates the potential for generation of patient-specific thyroid stem cells, which can be used for regenerative medicine and also lead to the generation of patient-specific cell lines that can potentially be used to model thyroid diseases and ultimately act as substrate for testing new therapeutic agents.

## Conflict of Interest Statement

The authors declare that the research was conducted in the absence of any commercial or financial relationships that could be construed as a potential conflict of interest.
